# Factors Associated With Low Physical Activity in Two Latin American Populations at Risk of Developing Type 2 Diabetes: An Exploratory Analysis

**DOI:** 10.3389/fpubh.2020.589484

**Published:** 2021-01-14

**Authors:** Tania Acosta, Rafael Tuesca, Karen Florez, Noël C. Barengo, Luis Anillo, Victor Flórez-García, Jorge Acosta, Liliana Carvajal, Sandra de la Rosa, María Julieta Pachón, Pablo Aschner

**Affiliations:** ^1^Departamento de Salud Pública, Universidad del Norte, Barranquilla, Colombia; ^2^Departamento de Matemáticas y Estadística, Universidad del Norte, Barranquilla, Colombia; ^3^Department of Translational Medicine, Herbert Wertheim College of Medicine, Florida International University, Miami, FL, United States; ^4^Department of Public Health, Faculty of Medicine, University of Helsinki, Helsinki, Finland; ^5^Riga Stradins University, Riga, Latvia; ^6^Joseph J. Zilber School of Public Health, University of Wisconsin – Milwaukee, Milwaukee, WI, United States; ^7^Asociación Colombiana de Diabetes, Bogotá, Colombia; ^8^Javeriana University, Bogotá, Colombia; ^9^San Ignacio University Hospital, Bogotá, Colombia

**Keywords:** physical activity, risk factors, population, pre-diabetic state, Latin America

## Abstract

Low level of physical activity is a risk factor for chronic non-communicable diseases. Specifically, people at risk of Type 2 Diabetes (T2D) have shown to benefit from being physically active. The objective of this study was to explore what factors were associated with low physical activity in people at high risk of T2D living in Bogota and Barranquilla, Colombia.

**Methodology:** Cross-sectional study using baseline data from a quasi-experimental clinical trial (PREDICOL Project). The study included 1,135 participants of Bogota and Barranquilla that presented a high risk of developing T2D according to the Finnish Diabetes Risk Score (>12 points) and who underwent an oral glucose tolerance test. The main outcome variable was the level of physical activity assessed by the International Physical Activity Questionnaire. Unadjusted and adjusted logistic regression analysis were used to calculate odds ratios (OR) and the corresponding 95% confidence intervals (CI).

**Results:** In total, 72.5% of the study participants had low level of physical activity. Participants in the age group between 45 and 54 years showed 74% greater odds of having low physical activity compared with the youngest age group (OR 1.74, 95% CI 1.1 −2.8). People living in Barranquilla were eight times more likely to have low physical activity compared with those in Bogotá D.C. (OR 8.1, 95% CI 5.7 to 11.4).

**Conclusion:** A large proportion of the population at risk of developing D2T in two large cities of Colombia have a sedentary lifestyle. Interventions should be designed and implemented in order to increase physical activity in these populations.

## Introduction

Globally, the prevalence of low physical activity varies from 16.3% in Oceania to 39.1% in Latin America (1). The Pan-American Health Organization estimated that three-quarters of the adult population is sedentary (2). Previous studies have suggested that the differences in the prevalence of low physical activity in the Americas are due to different methods of measurement (3–5). The prevalence of low physical activity in Colombia varies between 24, 4–79% (3, 5, 6). Furthermore, people between 30 to 49 years-old present double the probability of low activity physical, to live in low socioeconomic, and perceive themselves with a perception of regular or poor health (3).

There is consistent scientific evidence on the benefits of physical activity on overall health (3, 7–9). Specifically, previous studies have shown that people with low levels of physical activity have an increased risk of chronic non-communicable diseases (CNCD) (10–12). Also, low physical activity has been associated with lower survival in these patients T2D in low and middle-income countries (10, 11). Finally, low levels of physical activity is estimated to be strongly associated with insulin resistance and cardiovascular risk factors (13, 14).

Previous studies in Colombia revealed that people at risk of T2D had a higher prevalence of sedentary lifestyle compared with people with normal glucose tolerance (1, 9, 15). Similar results have been reported in studies targeting lifestyle behavior in people at high risk of T2D (16, 17) reporting lower levels if physical activity in populations with either impaired glucose tolerance (IGT) or impaired fasting glucose (IFG) compared with individuals with a healthy glucose metabolism.

Several determinants have been related to physical inactivity in the population. Low socioeconomic status have shown to be associated with low physical activity levels (1, 3, 7). Furthermore, several factors such as lack of time, resources, and being a woman have been linked with physical inactivity in Latin America (3, 4, 7, 18). Also extended periods of watching TV, having reduced the number of hobbies as well as not spending enough time outdoors besides, being overweight and obese have been identified as predictors of low physical activity (4, 10). Moreover, the type of built environment in cities is also an important factor contributing to physical activity levels in the population. Factors such as a neighborhood, the availability of space in an urban area, environmental planning, perceived safety, and road safety influence the practice of physical activity (4, 11, 14, 18).

However, information on determinants of low physical activity in people at risk of T2D in Colombia is poor. The objective of this study was to explore what factors were associated with low physical activity in people at high risk of T2D in Bogota and Barranquilla.

## Materials and Methods

### Study Design and Population

This is a cross-sectional study using baseline data from PREDICOL, a clinical trial designed to evaluate a community health program for the prevention of T2D and other cardiometabolic risk factors in the adult population of two districts in Bogotá (the capital city of Colombia) and Barranquilla (the main Atlantic port city) (PREDICOL Project; ClinicalTrials.gov identifier NCT03049839).

The study participants were recruited from 1350 households of lower socio-economic population strata in Bogota and Barranquilla. All persons older than 30 years-of-age in each household were asked to fill in the Finnish Diabetes Risk Score (FINDRISC) (19, 20). Each respondent with a FINDRISC >12 was invited to an oral glucose tolerance test (OGTT; *n* = 2,118). The threshold of FINDRISC> 12 was chosen according to the recent guidelines for screening for people at high risk of T2D released by the Colombian Ministry of Health and Social Protection (21). Finally, the results of the OGTT were obtained from 1,176 household members. Pregnant women and people with T2D were excluded in this study as well as people with missing data on the variables to be used in this analysis. The final study sample included 1,135 participants.

### Physical Activity Assessment

Physical activity was assessed using the short version of the International Physical Activity Questionnaire (IPAQ-SV) which has been validated in many countries including in Latin America (22–25). This version consists of seven questions assessing the frequency, intensity, and duration of physical activity during the last 7 days. The questionnaire also includes the time during which the person remains seated. The data obtained allows to calculate metabolic equivalents of task (METs) per minutes during a regular week. The selected MET values were derived from work undertaken during the IPAQ Reliability Study undertaken in 2000–2001 (24). Using the Ainsworth et al. Compendium an average MET score was derived for each type of activity (26). For example, all types of walking were included and an average MET value for walking was created. The same procedure was undertaken for moderate-intensity activities and vigorous-intensity activities. The following values continue to be used for the analysis of IPAQ data: Walking = 3.3 METs, Moderate PA = 4.0 METs and Vigorous PA = 8.0 METs. Using these values, four continuous scores were defined: Walking MET-minutes/week = 3.3 ^*^ walking minutes ^*^ walking days; Moderate MET-minutes/week = 4.0 ^*^ moderate-intensity activity minutes ^*^ moderate days; Vigorous MET-minutes/week = 8.0 ^*^ vigorous-intensity activity minutes ^*^ vigorous-intensity days. And (iv) total physical activity MET-minutes/week = sum of Walking + Moderate + Vigorous MET minutes/week scores. The participants were then categorized into low physical activity (≤ 479 METs-min/week), moderate physical activity (480–1,499 METs-min/week) and high physical activity (≥ 1,500 METs-min/week), accordingly. Also, participants were asked whether they perform routinely at least 30 min per day of physical activity at work and/or leisure time.

Physical activity was also self-reported in the FINDRISC questionnaire. The FINDRISC score was developed by the researchers of the Diabetes prevention study in Finland (DPS) (19, 20). It has been adapted and validated to predict diabetes in the Latin America region. The pertinent question asks whether the subject practices regularly at least 30 min of daily physical activity at work or during leisure time. The questionnaire also assessed daily fruit and vegetable consumption, current use of hypertension medication, family history of T2D and whether the subject has had a past history of hyperglycemia.

### Measurements

Height and weight were measured without shoes and with light clothing. BMI was calculated as weight (kg) divided by height^2^ (m^2^). Waist circumference (to the nearest cm) was measured at the approximate midpoint between the lower margin of the last palpable rib and the top of the iliac crest. Blood pressure (2 mmHg precision) was be recorded twice while the study participants remained in a seated position. The mean value of the two measurements was used for this analysis. All participants underwent an OGTT that was carried out according to the World Health Organization (WHO) recommendations (27). The test started after at least 8 h fasting, and the 2-h blood samples was obtained after oral ingestion of water solution with 75 g anhydrous glucose. The glucose tolerance status was classified according to the criteria of the World Health organization (28, 29). Individuals who had fasting plasma glucose (FPG) level ≥126 mg/dl or 2 h plasma glucose (2hPG) ≥200 mg/dl were classified as having T2D. Those with 2hPG≥140 mg/dl but <200 mg/dl, and FPG <126 mg/dl were classified as having impaired glucose tolerance (IGT). Impaired fasting glucose (IFG) was defined as FPG ≥110 but <126 mg/dl, and 2hPG <140 mg/dl. In all cases, a supervisor field was trained to check all the information collected, this didn't allow missing information in the dataset.

### Ethical Issues

The study was approved by the ethics committee of the Universidad del Norte through the 141-evaluation act. April 28th, 2016. All participants signed the consent form and could withdraw from the study at any moment. The study followed the norms of good clinical practice and the Helsinki guidelines.

### Statistical Analysis

Data processing and analysis were performed in the statistical software SPSS version 25 (SPSS; Chicago, IL, USA). Distributions of physical activity frequencies are presented in absolute numbers and percentages. Chi-square tests were used to evaluate the differences in proportions between the subgroups. Continuous variables are summarized in medians and interquartile ranges, after checking the normality through the Kolmogorov–Smirnov test. To examine the relationship between the level of physical activity and some baseline characteristics of the participants, non-parametric estimates for ordinal variables were used (Spearman and Kendall tests). Collinearity diagnostics were performed to assess the assumptions of the model. To evaluate the factors associated with low physical activity, binary logistic regression models were used. Total physical activity was dichotomized into (i) sufficient physical activity (moderate or high physical activity and (ii) low physical activity (none or low physical activity). First, unadjusted logistic regression models were performed to identify covariates that were associated with low physical activity. Then, a backward logistic regression model was used including all variables that had a statistically significant association with low physical activity in the unadjusted logistic regression analysis. Variables were removed from the model according to Wald probability statistics. Odds ratios (OR) and 95% confidence intervals (95% CI) were calculated. A *p*-value of <0.05 was considered statistically significant.

## Results

Data from 1,135 participants were included (Table 1). Three out of four participants were female. The prevalence of having high levels of physical activity was higher in both men and women living in Bogota than in Barranquilla. Whereas, 16.8% of men and 13% of women were physical active in Bogota, the corresponding prevalence was 8.4% (men) and 1.6% (women) in Barranquilla. More than half of the participants had only elementary school or no education. In men, there were no statistically significant differences in educational levels, smoking status, BMI, fruit intake, receiving hypertension treatment or glucose metabolism disorders according to the physical activity categories. However, a higher proportion of men 55 years-of-age and older engaged in high physical activity compared with the younger age-groups (*p*-value 0.017). In women, those with lower education had a higher percentage practicing high physical activity compared with women with higher education (*p*-value 0.028). No statistically significant differences were observed in the distribution of smoking, age groups, BMI, daily fruit intake, hypertension treatment and glucose metabolism disorders.

**Table 1:** Baseline characteristics of the participants of PREDICOL according to their physical activity levels at baseline and sex.

**Variable**	**Men**			***p*-value^***a***^**	**Women**			***p*-value^***a***^**
	**Physical activity**				**Physical activity**			
	**Low**	**Moderate**	**High**		**Low**	**Moderate**	**High**	
	**193 (72.0)**	**42 (15.7)**	**33 (12.3)**		**661 (76.2)**	**144 (16.6)**	**62 (7.2)**	
**City**								
Barranquilla	124 (86.7)	7 (4.9)	12 (8.4)	<0.001	414 (93.2)	23 (5.2)	7 (1.6)	<0.001
Bogota D.C.	69 (55.2)	35 (28.0)	21 (16.8)		247 (58.4)	121 (28.6)	55 (13.0)	
**Education level**								
No schooling	47 (68.1)	12 (17.4)	10 (14.5)	0.941	151 (69.3)	45 (20.6)	22 (10.1)	0.028
Elementary school	79 (71.8)	17 (15.5)	14 (12.7)		278 (76.2)	59 (16.2)	28 (7.7)	
Junior high school	44 (77.2)	7 (12.3)	6 (10.5)		157 (82.6)	23 (12.1)	10 (5.3)	
Superior	23 (71.9)	6 (18.8)	3 (9.4)		74 (79.6)	17 (18.3)	2 (2.2)	
**Current smokers**								
Yes	24 (68.6)	4 (11.4)	7 (20.0)	0.295	34 (64.2)	14 (26.4)	5 (9.4)	0.091
**Age groups (years)**								
<45	27 (77.1)	5 (14.3)	3 (8.6)	0.017	145 (75.9)	36 (18.8)	10 (5.2)	0.069
45-54	49 (83.1)	7 (11.9)	3 (5.1)		189 (82.2)	27 (11.7)	14 (6.1)	
55-64	61 (75.3)	7 (8.6)	13 (16.0)		191 (74.3)	41 (16.0)	25 (9.7)	
> 64	56 (60.2)	23 (24.7)	14 (15.1)		136 (72.0)	40 (21.2)	13 (6.9)	
**BMI (kg/m**^**2**^**)**								
Normal	27 (69.2)	5 (12.8)	7 (17.9)	0.519	78 (77.2)	16 (15.8)	7 (6.9)	0.672
Overweight	91 (70.0)	21 (16.2)	18 (13.8)		250 (74.2)	64 (19.0)	23 (6.8)	
Obesity	75 (75.8)	16 (16.2)	8 (8.1)		333 (77.6)	64 (14.9)	32 (7.5)	
**Daily fruit intake**								
No	174 (72.2)	39 (16.2)	28 (11.6)	0.510	558 (76.3)	126 (17.2)	47 (6.4)	0.105
Yes	19 (70.4)	3 (11.1)	5 (18.5)		103 (75.7)	18 (13.2)	15 (11.0)	
**Physical activity**								
<30 min physical activity/d	178 (73.9)	37 (15.4)	26 (10.8)	0.055	617 (77.9)	121 (15.3)	54 (6.8)	0.001
≥30 min physical activity/d	15 (55.6)	5 (18.5)	7 (25.9)		44 (58.7)	23 (30.7)	8 (10.7)	
**Treatment Hypertension**								
No	116 (75.3)	21 (13.6)	17 (11.0)	0.371	415 (76.3)	92 (16.9)	37 (6.8)	0.848
Yes	77 (67.5)	21 (18.4)	16 (14.0)		246 (76.2)	52 (16.1)	25 (7.7)	
**Oral glucose tolerance test (OGTT)**								
NT	132 (71.7)	32 (17.4)	20 (10.9)	0.697	502 (75.3)	116 (17.4)	49 (7.3)	0.520
IFG	6 (66.7)	1 (11.1)	2 (22.2)		19 (70.4)	4 (14.8)	4 (14.8)	
IGT	39 (76.5)	6 (11.8)	6 (11.8)		78 (82.1)	12 (12.6)	5 (5.3)	
T2D	16 (66.7)	3 (12.5)	5 (20.8)		62 (79.5)	12 (15.4)	4 (5.1)	

a *Test of homogeneity (X^2^); n (%). BMI, body mass index; NT, Normotolerence; IFG, impaired fasting glucose; IGT, impaired glucose tolerance; T2D, type 2 diabetes*.

No statistically significant differences were observed between sitting time, BMI, fasting glucose, 2-h glucose, diastolic blood pressure and the prevalence of low, moderate, and high physical activity (Table 2). However, systolic blood pressure was statistically significantly and inversely correlated with physical activity (*r* = −0.069 and $\bar{\text{C}}$=-0.065). Whereas, 40% of the study participants with high physical activity had systolic blood pressure levels of >140 mmHg, the corresponding prevalence of high systolic blood pressure was only 28% in the moderate physical activity group and 24.4% in the low physical activity group (*p* < 0.05). No statistically significant differences were found between sitting time and low, moderate and high physical activity (*r* = −0.013, $\bar{\text{C}}$=-0.012; *p* > 0.05).

**Table 2:** Characteristics of study participants of PREDICOL according to their level of physical activity.

**Variable**	**Classification physical activity**			**Rank correlation**	
	**Low**	**Moderate**	**High**	***r***	**$\bar{\text{C}}$**
	**(*n* = 854)**	**(*n* = 186)**	**(*n* = 95)**		
**Sitting time (minutes/week)**					
≤ 420	525 (61.5)	115 (61.8)	64 (67.4)	−0.013	−0.012
421–840	148 (17.3)	23 (12.4)	14 (14.7)		
> 840	181 (21.2)	48 (25.8)	17 (17.9)		
**BMI (kg/m**^**2**^**)**					
≤ 24.9	105 (12.3)	21 (11.3)	14 (14.7)	−0.038	−0.036
25–29.9	341 (39.9)	85 (45.7)	41 (43.2)		
> 29.9	408 (47.8)	80 (43.0)	40 (42.1)		
**Fasting glucose (mg/dl)**					
≤ 109	741 (86.8)	163 (87.6)	80 (84.2)	0.006	0.005
110–125	66 (7.7)	14 (7.5)	10 (10.5)		
> 125	47 (5.5)	9 (4.8)	5 (5.3)		
**2-h glucose levels (mg/dL)**					
≤ 139	687 (80.4)	157 (84.4)	78 (82.1)	−0.031	−0.030
140–199	121 (14.2)	20 (10.8)	11 (11.6)		
> 199	46 (5.4)	9 (4.8)	6 (6.3)		
**Systolic blood pressure (mmHg)**					
≤ 135	577 (67.6)	123 (66.1)	51 (53.7)	−0.069*	−0.065*
136–140	69 (8.1)	11 (5.9)	6 (6.3)		
> 140	208 (24.4)	52 (28.0)	38 (40.0)		
**Diastolic blood pressure (mmHg)**					
≤ 84	495 (58.0)	102 (54.8)	43 (45.3)	−0.054	−0.051
85–89	136 (15.9)	34 (18.3)	21 (22.1)		
> 89	223 (26.1)	50 (26.9)	31 (32.6)		

n (%); *Correlation is statistically significant at the 0.05 level; r, Spearman rank correlation. $\bar{\text{C}}$, Kendall rank correlation.

Low physical activity assessed by the IPAQ-SV as the reference point (gold standard). A negative answer to the question on physical activity in the FINDRISC had a very high sensitivity (93%; 95% CI 91–95%) but a very low specificity (15%; 95% CI 11–20%) compared with low physical activity assessed by the IPAQ-SV (data not shown). The positive predictive value was 77% (95% CI 76–78%) and the negative predictive value was 42% (95% CI 34–51%). The concordance between the two scores was also very poor (kappa 0.11). Nevertheless, people who reported <30 min of daily physical activity during work and/or leisure time in the FINDRISC had more than twice the probability of showing a low physical activity in the IPAQ-SV (OR 2.4; 95% CI 1.6-3.7).

Table 3 shows the unadjusted and adjusted odds ratios of the determinants of low physical activity in the participants of PREDICOL at baseline. Age group and city of residence was all statistically significantly associated with low physical activity levels in the adjusted logistic regression analysis. Participants in the age group between 45 and 54 years showed 74% greater odds of having low physical activity compared with the youngest age group (OR 1.74, 95% CI 1.1 −2.8). People living in Barranquilla were eight times more likely to have low physical activity compared with those in Bogotá D.C. (OR 8.1, 95% CI 5.7 to 11.4).

**Table 3:** Risk factors associated with low physical activity at baseline of the PREDICOL.

**Variable**	**Low physical activity**	
	**OR (95% CI)**	**OR (95% CI)^**∫**^**
**Age groups (years)**		
<45	Ref.	Ref.
45-54	1.46 (0.95–2.25)	1.74 (1.1–2.8)
55-64	0.92 (0.62–1.36)	1.2 (0.81–1.92)
> 64	0.67 (0.45–0.99)	1.0 (0.67–1.62)
**Sex**		
Male	Ref.	Ref.
Female	1.25 (0.92–1.7)	1.33 (0.94–1.9)
**City**		
Bogotá D.C.	Ref.	Ref.
Barranquilla	8.1 (5.7–11.3)	8.1 (5.7–11.4)
**Education level**		
No schooling	Ref.	
Elementary school	1.36 (0.98–1.88)	
Junior high school	1.96 (1.31–2.95)	
Superior	1.55 (0.95–2.54)	
**Body Mass Index (kg/m**^**2**^**)**		
<25 (kg/m^2^)	Ref.	
≥ 25 (kg/m^2^)	1.01 (0.67–1.52)	
**Current smokers**		
No	Ref.	
Yes	0.61 (0.38–0.97)	

∫ *The adjusted model included age group, sex and city; OR, Odds Ratio; Ref, reference group*.

## Discussion

Our data revealed that study participants in Barranquilla were more likely to have low levels of physical activity compared with people at risk of T2D in Bogota. Furthermore, age between 45 and 54 years-old was associated with increased probability of low physical activity compared with younger people (age 30–45 y). No differences were found in sitting time, diastolic blood pressure, BMI or blood glucose levels according to physical activity levels.

The fact that three out of four participants at higher risk of developing T2D had low levels of physical activity is of concern. General population studies have shown that the prevalence of physical inactivity is higher in low and middle-income countries compared to high-income countries (1). In the ELAN study carried out in eight Latin American countries, the overall prevalence of physical inactivity was 41%, ranging between 27% in Chile to 47% in Costa Rica and Venezuela. The prevalence was higher in women and people with more education (30). Moreover, a study of adolescents from Latin America and the Caribbean showed that around 15% were physically active (moderate to vigorous for at least 1 h/day) (24). It should be noted that the prevalence of physical inactivity found in Bogotá is similar to that reported in western high-income countries, while Barranquilla was similar to that reported for Kuwait (1). These differences are probably related to urban design, climatic conditions, and behavioral aspects (1). In Colombia, a previous study conducted in Barranquilla (DEMOJUAN) also revealed a high prevalence of physical inactivity (around 80%) (15). Furthermore, they also observed a higher proportion of sedentary lifestyles in those individuals at high risk of type 2 diabetes (1, 9). Likewise, it has been reported that men carry out more moderate and intense physical activity than women. A study carried out in Bogotá found an OR 1.62 (95% CI: 1.31 to 2.01) in men compared to women for these levels of physical activity (30). It is important to indicate that the time that an individual is sitting does not necessarily correlate with the level of physical activity performed. A study from the Medical University of Lodz showed that the time that students remained seated was an average of 46 h per week, but 65% were in the high category of physical activity and <2% were in a low category, using the long version of the IPAQ (9). In the PREDICOL study we found that most of the studied population does not follow a healthy lifestyle (physical inactivity, low intake of fruits and vegetables and high prevalence of overweight and obesity). Similar results have been reported in diabetes prevention studies conducted in Spain, Poland, Greece and Finland (16, 17). Comparing the National Surveys on food intake and nutrition (ENSIN) conducted in Colombia in 2010 and 2015 there was a significant decrease in physical activity (at least 150 min/week) from 53.5 to 51.1% (31).

The adjusted prevalence of people in a study who carried out regular physical activity in Bogotá was 36.8%, this figure was similar to our findings in the same city (30). The causes can be multiple, including different facilities and local incentives. Bogotá, for example, has been implementing an intense PA policy by building large recreational parks and playgrounds, as well as an extensive network of urban bike trails. There are also different climatic conditions with Barranquilla having a very hot and humid climate while Bogotá is located in a plateau with temperate climate all year round. In a longitudinal data study of individuals ages 18 to 55 in the 1991–2006 China Health and Nutrition Surveys, there was a 32% decrease in average weekly physical activity. This was strongly associated with urbanization factors such as greater availability of higher education institutions, housing infrastructure, sanitation improvements, and the economic well-being of the community in which people operate (32). In our case, Barranquilla has been experiencing intense economic and cultural development for the last decades and is now the most important city in the Caribbean coast. Similarly, it is important to note that Colombia is a heterogeneous country, where habits are markedly different in each territory. Bogotá and Barraquilla belong to the group of the 5 largest cities in Colombia and our study was carried out in urban environments, so the conclusions of this research could not be extrapolated to smaller populations or rural areas; making this a potential source of research for future projects. Additionally, the use of urban space built for pedestrians (sidewalks, parks) has been one of the main priorities in the last decades in Bogotá, but less so in Barranquilla.

Our study has some limitations. The use of questionnaires do not provide as precise data on physical activity as could be provided by direct measurements such as the use of pedometers. Most of the participants were below the high school level, so the final results cannot be extrapolated to all communities. The cross-sectional design of this analysis may limit inferences on the causal relationship of risk factors with low levels of physical activity. There may be important variations in physical activity levels according to household levels and thus, an overall effect could mask lower level variations at the level of household. All study participants of the two cities included in the study belong to the lowest two out of six socio-economic levels. In addition, there may be differences in physical activity levels according to living setting (such as urban or rural areas) as well we could not account for. Thus, our results do not allow to generalize to populations of higher household, respectively, socio-economic levels or different living settings. Therefore, the findings of our study should be considered for exploratory purposes only.

## Conclusion

A large proportion of the population at risk of developing T2D in two large cities of Colombia have a sedentary lifestyle. Moreover, the proportion of people at risk of T2D with low physical activity levels was higher in the districts of Barranquilla. Thus, our study provides more scientific evidence to support the need for lifestyle intervention programs targeting an increase of physical activity in people at high risk of T2D. The use of devices to measure physical activity directly such as pedometers and GPS tracking may provide more accurate data to correlate with environmental and lifestyle variables.

## Data Availability Statement

The datasets presented in this article are not readily available because The PREDICOL Project is a Project that is currently in progress, therefore the database according to the regulations may not be published until the end of the study, for any requirement you can contact the authors. Requests to access the datasets should be directed to tacosta@gmail.com.

## Ethics Statement

The studies involving human participants were reviewed and approved by ethics committee of the Universidad del Norte, Barranquilla, Colombia. The patients/participants provided their written informed consent to participate in this study.

## Author Contributions

PA, TA, RT, KF, NB, and JA contributed conception and design of the study. SR, TA, PA, LA, MP, and LC supervised data collection. LA organized the dataset. KF, TA, LA, PA, VF-G, and LA performed the statistical analysis. TA, RT, KF, VF-G, and LA wrote the first draft of the manuscript with support from PA and NB. All authors contributed to manuscript revision, read and approved the submitted version.

## Conflict of Interest

The authors declare that the research was conducted in the absence of any commercial or financial relationships that could be construed as a potential conflict of interest.

## Abbreviations

D2T: Type 2 Diabetes
WHO: World Health Organization
NCD: chronic non-communicable diseases
OGTT: glucose tolerance test.
